# Screening of Bioactivities and Toxicity of *Cnidoscolus quercifolius* Pohl

**DOI:** 10.1155/2016/7930563

**Published:** 2016-05-12

**Authors:** Paulo Fernando Machado Paredes, Fábio Roger Vasconcelos, Raquel Teixeira Terceiro Paim, Márcia Maria Mendes Marques, Selene Maia De Morais, Sandra Machado Lira, Isabel Desidério Braquehais, Ícaro Gusmão Pinto Vieira, Francisca Noelia Pereira Mendes, Maria Izabel Florindo Guedes

**Affiliations:** ^1^Northeast Biotechnology Network, Graduate Program of Biotechnology, State University of Ceará, Campus do Itaperi, 60714-903 Fortaleza, CE, Brazil; ^2^Phytopathology Laboratory, Embrapa Agroindústria Tropical (EMBRAPA), Federal University of Ceará, Campus do Pici, 60455-970 Fortaleza, CE, Brazil; ^3^Degree Course in Biological Sciences, Federal University of Piauí, Campus Universitário Ministro Petrônio Portella, 64049-550 Teresina, PI, Brazil; ^4^Course of Chemistry, State University of Ceará, Campus do Itaperi, 60714-903 Fortaleza, CE, Brazil; ^5^Biotechnology and Molecular Biology Laboratory, State University of Ceará, Campus do Itaperi, 60714-903 Fortaleza, CE, Brazil

## Abstract

The* caatinga*, an exclusively Brazilian biome, is one of the most endangered vegetation systems in the planet. To be exploited rationally, its potential needs to be scientifically demonstrated. Among these is the* faveleira*, used in northeastern Brazil. It stands out for its extraordinary drought resistance and medicinal properties. The objective of this study was to assess the therapeutic potential of compounds extracted from* Cnidoscolus quercifolius* Pohl in preventing disease and its rational use as a herbal therapeutic tool. The methodology began with the collection and herborization of the plant material, to obtain the chemical compounds, preliminary phytochemical analysis, and extraction of the constituents of the active extracts. To determine the biological activities the authors conducted investigation of antioxidant and antimicrobial activities, inhibition capacity of the acetylcholinesterase enzyme, and initial assessment of toxicity of the extracts. The results demonstrated great potential as an antimicrobial agent, an important antioxidant capacity, and acetylcholinesterase inhibition response with no significant difference compared with the reference drug. The authors expect to develop a new herbal product, resulting in lower production costs and that, consequently, could be commercialized in more accessible form to the population, highlighting the risk reduction of contraindication of this category of medications.

## 1. Introduction

The importance of medicinal plants in solving the health problems of the world is gaining more and more attention. Due to this increased interest, research on plants of medicinal importance is growing phenomenally at the international level. Moreover, a growing number of people are seeking traditional medicine for their primary health care.

Man has long been using plants to promote health, in the belief that they carry beneficial substances for a healthy existence. Currently, this practice constitutes an amount of internalized knowledge shared among multiple users or in more traditional communities [1]. In Brazil, the first Europeans who settled in the land ran across a large quantity of medicinal plants used by the indigenous populations who lived here. It was the start of a series of cultural intermixes that blended the knowledge brought in by the colonizers, the slaves, and the natives. Today, according to Amorozo [2], the introduction of modern medicine offers another option for health practices in traditional communities that use popular medicine. In many cases, the procedures of modern medicine and popular medicine are supplemental and constitute an effective form of primary health care, which may supplement the treatment usually employed in the lower income population.

According to López [3], the common knowledge on the use and effectiveness of medicinal plants contributes significantly to the dissemination of the therapeutic properties of plants that are frequently used, although their chemical constituents and main active principles are not completely known.

The World Health Organization (WHO) has recognized and recommended the dissemination of traditional knowledge and use of phytotherapic medicines. For this, the organization launched the document entitled “National Policy on Traditional Medicine and Regulation of Herbal Medicines” which discusses the policies for use of the genetic vegetal diversity [4]. This document aims to ensure secure access to phytotherapic remedies, considering traditional knowledge on medicinal plants, as well as promote research and encourage the development of technology and innovation, stimulating the use of medicinal plants in different stages of the production chain, ultimately fostering the sustainable use of biodiversity.

In Brazil, the pharmaceutical industry grossed an estimated US$ 8 billion in revenues in 1996, 25% of which originated from medicines derived from plants [5], and in the latest national balance, this market closed with a revenue of US$ 68 billion [6]. The United States and Germany rank among the biggest consumers of natural products from Brazil. Between 1994 and 1998, the USA and Germany imported, respectively, 1,521 and 1,466 tons of plants that are usually shipped to those countries under the generic label of “plant material from Brazil,” according to the Brazilian Institute of Environment and Renewable Natural Resources [7]. Despite the largest natural reservoir of plant diversity in the planet, only 8% of this immense wealth has been studied in research of bioactive compounds, and roughly 1,500 species have been evaluated in their medicinal properties [5].

Spreading across an area of approximately 850 thousand square km, the caatinga, an exclusively Brazilian biome, is also one of the most endangered areas in the planet. Despite this unique condition, there was not enough incentive to direct local preservation botanical studies [8]. Several authors call attention to the direct dependency on this environmental resource by the populations living out of extractivist activities within the biome [9, 10]. The semiarid region of the Brazilian Northeast is known for hosting a number of native species that are under systematic exploitation in recent years, without any sustainable management, which ultimately causes the extinction of some species, thus unleashing a rural exodus. Thus, the therapeutic potential of native plants must be scientifically demonstrated, so that it can be exploited in a rational and orderly manner [11, 12].

The* Cnidoscolus quercifolius* (Pohl) popularly known as “favela” or “faveleira” is a forager that is part of the native species from the caatinga, commonly found in all the states of northeastern Brazil. It is widely used as fodder for cattle and small ruminants, mainly in the dry periods. It is a rustic plant of rapid growth, belonging to the Euphorbiaceae family [13] and* Cnidoscolus* genus [14]. Its xerophile character allows the plant to grow and reproduce, even in periods of prolonged droughts, thus contributing to keep the balance of the ecosystem, mitigating environmental degradation. According to Ribeiro and Brito [15], the faveleira can be used for forestation, recovery of degraded areas, animal and human consumption, medicine, timber, and energy.

Most studies on the faveleira are targeted at its use in the agricultural and livestock fields, and very few studies of the species focus on the area of chemistry of natural products and biological activity. For this reason, the present study focuses on the assessment of the therapeutic potential of this plant, by investigating its possible antioxidant, antimicrobial, and inhibitory activity of the acetylcholinesterase enzyme (AChE), as well as the toxicity of the methanolic extracts from three parts of* Cnidoscolus quercifolius* Pohl.

## 2. Materials and Methods

### 2.1. Plant Material

*Cnidoscolus quercifolius* was collected from its natural habitat in the city of Fortaleza, Ceará (northeastern Brazil), and identified by a botanist of the Prisco Bezerra Herbarium (Federal University of Ceará, Brazil), where a voucher specimen was deposited (reference number 56043).

### 2.2. Preparation of Extracts

Different plant tissues (leaves, roots, and roots barks) were powdered and subjected to extraction with methanol at room temperature (25–28°C) for seven days. The solution was filtered through paper filter and evaporated in a rotary evaporator under reduced pressure, obtaining yields 36,7% (leaves methanolic extract (LME)), 7,47% (root methanolic extract (RME)), and 7,82% (root bark methanolic extract (RBME)).

### 2.3. Preliminary Phytochemical Analysis

The extracts were subjected to phytochemical screening, following the protocols described by Mattos [16]. Chemical tests were performed using specific reagents, observing color changes or formation of precipitate, characteristic for each class of substances. Tests were performed for the detection of phenols and tannins, anthocyanins and anthocyanidins, flavones, flavonols, xanthones, flavanones, chalcones and aurones, leucoanthocyanidins, catechins, steroids and triterpenoids, and saponins.

### 2.4. Antioxidant Activity

The* in vitro* antioxidant activity of the extracts was determined using the 1,1-diphenyl-2-picrylhydrazyl (DPPH) method, as previously reported by Brand-Williams et al. [17], with some modifications. Different dilutions of the extracts (5–10.000 ppm in methanol) were prepared in triplicate. The sample diluted extracts (0,1 mL) were mixed with 3,9 mL of methanol solution containing DPPH (6,5 × 10^−5^ mol·L^−1^) for 1 h. The reduction of the DPPH radical was measured by continuously monitoring the decrease in absorption at 515 nm. Antioxidant activity is given as the percentage of DPPH radical scavenged which was calculated according to the equation % = [(Abs_DPPH_ − Abs_sample_)/Abs_sample_] × 100, where Abs_DPPH_ is the absorbance of the DPPH solution and Abs_sample_ is the absorbance of the sample extract. Results were expressed as IC_50_ concentration where 50% inhibition of the DPPH radical is obtained.

### 2.5. Antimicrobial Screening

The authors used the strains of* Enterococcus faecalis* ATCC 19433,* Pseudomonas aeruginosa* ATCC 10145,* Klebsiella pneumoniae* ATCC 13883,* Escherichia coli* ATCC 11775,* Staphylococcus epidermidis* ATCC 12228, and* Enterococcus faecium* ATCC 51559 from the Oswaldo Cruz Foundation (FIOCRUZ), Rio de Janeiro, Brazil, and the fungi* Fusarium* sp. LF48,* F. solani* LF04,* Fusarium solani* LF104,* Lasiodiplodia theobromae* LF11,* Lasiodiplodia theobromae* LF124,* Lasiodiplodia theobromae* LF126, and* Colletotrichum gloeosporioides* LF50, from the Phytopathology Laboratory of Embrapa Agroindústria Tropical (EMBRAPA), Fortaleza, CE.

The preliminary antibacterial tests were conducted on concentrations of 25 and 250 mg·mL^−1^, in accordance with Bauer et al.'s methodology [18]. Disks containing Streptomycin (1.0 mg·mL^−1^) and Chloramphenicol (4.0 mg·mL^−1^) were used as positive control.

To determine the MIC (Minimum Inhibitory Concentration), the authors used a dilution technique in microplates of 96 wells, where the wells were filled with 100 *μ*L of Müller-Hinton broth plus 100 *μ*L of the extracts solutions [19]. Then they performed a serial dilution of 2,500 at 4.88 *μ*g·mL^−1^. Bacterial strains were previously inoculated in agar Brain Heart Infusion (BHI) at 35°C for 18 h standardized at a scale of 0.5, McFarland, in saline solution 0.85%, followed by 1 : 20 dilution, obtaining values of 10^5^ to 10^6^ cells·mL^−1^ [20], and added in each orifice of the microplates (10 V/V). Chloramphenicol (4.0 mg·mL^−1^) was used as positive control. The microplates were incubated in an incubator (Quimis, Q316M4), at 37 ± 2°C for 24 hours. A spectrophotometer was used (Bio-Rad, 550) after incubation, for reading of absorbance with a wavelength of 595 nm. Following the MIC test the authors collected 10 *μ*L from all the wells presenting no bacterial growth and the material was transferred to a Petri dish containing antibiotic-free Müller-Hinton agar, in order to verify cell viability (MBC).

For evaluation of fungicidal activity, the fungi were sown with Potato Dextrose Agar (PDA), incubated at 28 ± 2°C for 7 days. The mycelia were transferred to 125 mL Erlenmeyer flasks containing Tween 80 solution (0.1) and glass beads and shaken vigorously. They were filtered and centrifuged at 3000 rpm for 15 minutes and rinsed 3 times with Tween 80 (0.1%) for separation of spores. Dilutions were performed until reaching the concentration of 10^5^ to 10^6^ spores/mL. Then, they were distributed in 100 *μ*L of Roswell Park Memorial Institute (RPMI) medium in a microplate with 96 wells, added with 100 *μ*L of faveleira extracts, where serial dilutions (1 : 2 V/V) were carried out. The 100 *μ*L residual waste was discarded. A commercial fungicide was used (TECTOR® RC-Thiabendazole, Syngenta; 4.0 ML/L) as positive control. Finally, 50 *μ*L of fungal suspension was added to all wells, except for the lines intended for the sterilization control. The determination of the Minimum Inhibitory Concentration of Fungi (MIC-f) was obtained with a stereoscope, verifying the lower concentration of samples capable of inhibiting the growth of the microorganism, after 5 days of incubation. For the Minimum Fungicidal Concentration (MFC), the wells were divided at 50 *μ*L without fungal growth and inoculated in BDA and checked during 5 days.

### 2.6. Inhibition Test of Acetylcholinesterase (AChE)

TLC evaluated inhibition of acetylcholinesterase enzyme (AChE), in accordance with the methodology described by Ellman et al. [21], which was later adapted by Rhee et al. [22], following the protocols described by De Morais et al. [23].

5.5′-Dithiobis-2-nitrobenzoic acid (DTNB) solutions were used, and acetylcholine iodide (ATCI) was used in buffer. The authors used a MERCK TLC silica gel 60 F254 for sample application. The solvent was evaporated and, shortly thereafter, they pulverized the substrate (ATCI, 1 mM in buffer) and the Ellman reagent (DTNB, 1 mM in buffer), respectively. After 3 minutes the plate was sprayed with the enzyme AChE (3 U·mL^−1^) [23, 24]. The inhibition was indicated by the presence of white halos. The halos were measured and compared with the halo formed by physostigmine, a standard alkaloid.

### 2.7. *Brine Shrimp* Lethality Test

The lethality assay against* Artemia* sp. was carried out according to an adapted method proposed by Meyer et al. [25]. The* Artemia* sp. eggs were incubated in salt water at room temperature for a period of 48 hours in a glass vessel fitted with a dark compartment and another with receiving artificial light. With the aid of a light source, the hatched larvae were attracted to the lightened vessel part and were collected with a Pasteur pipette and then transferred to a beaker with saline water. Various extracts concentrations, previously dissolved in DMSO, were prepared in saline water ranging from 10,000 to 1 *μ*g·mL^−1^. The various solutions (5 mL) were added to plastic cups (50 mL) containing 10 larvae and the control containing only the diluent (DMSO and saline). Assays were performed in triplicate, and the number of dead larvae was counted after 24 hours.

## 3. Results and Discussion

The phytochemical analysis of methanolic extracts (LME, RME, and RBME) of* C. quercifolius* Pohl revealed the presence of phenols, tannins, flavones, flavonols, and xanthones (Table 1).

Peixoto Sobrinho et al. [26] carried out a phytochemical survey with four species of the genus* Cnidoscolus*. In the analysis of the leaves of* C. quercifolius,* they detected the presence of anthocyanins, anthracene derivatives, anthraquinones, flavonoids, tannins, coumarins, lignans, triterpenes, steroids, xanthines, monoterpenes, and diterpenes. The extract from the bark of* C. quercifolius* presented coumarins, saponins, steroids, monoterpenes, diterpenes, and triterpenes. In the bark of* C. quercifolius* De Araújo Gomes et al. [27] found coumarins, flavonoids, monoterpenes/diterpenes, and naphthoquinones, and in the leaves, they found coumarin compounds, anthracene derivatives, flavonoids, lignans, and triterpenes/steroids.

This study did not detect alkaloids in any of the* C. quercifolius* extracts, and a similar result was found by Peixoto Sobrinho et al. [26] and De Araújo Gomes et al. [27]. However, other species of the genus* Cnidoscolus* showed the presence of alkaloids [28–30].

This difference in the presence and absence of certain chemical compounds of plant species may be related to factors such as age of the plant and the time of collection [31]. Moreover, other factors such as seasonality, circadian rhythm, development, climatic factors, water availability, ultraviolet radiation, nutrients, air pollution, and altitude may influence the composition of the secondary metabolites of plants [32]. Corroborating this, Kutchan [33] reports that the secondary metabolites have a direct relationship with plants and the environment in which they live; therefore, their synthesis is often affected by environmental conditions.

Free radicals have their production controlled in living organisms by a number of endogenous antioxidants compounds or are derived from diet and supplements. Oxidative stress may occur when there is limitation in the availability of antioxidants. Thus, when there is presence of active antioxidant compounds within these systems, they are capable of stabilizing or deactivate free radicals before they strike the biological targets in the cells [34]. The excess of oxidative stress which leads to organic decay is mitigated or even prevented by antioxidants-based therapies. Thus, the antioxidant therapies used in a large number of illnesses related to the generation of free radicals, such as cancer, aging, atherosclerosis, ischemia, inflammation, and neurodegenerative diseases, seem to be promising [35].

After performing the test to evaluate the antioxidant potential of extracts of* C. quercifolius* it was found that the RBME presented better antioxidant result, with IC_50_ = 21.56 ± 0.71 *μ*g·mL^−1^, followed by LME with IC_50_ = 133.30 ± 0.73 *μ*g·mL^−1^ and RME with IC_50_ = 171.82 ± 0.69 *μ*g·mL^−1^, showing the presence of metabolites capable of sequestering free radicals. Quercetin was used as a standard, featuring IC_50_ = 5.0 ± 0.18 *μ*g·mL^−1^. These are samples of crude extracts and may therefore be considered a meaningful result, suggesting the need to isolate the compounds responsible for sequestering the free radicals, thus achieving an index even closer to the default. As far as we know, there are no data in the literature showing the antioxidant activity of samples of* C. quercifolius*; therefore, these are unpublished data.

Studies conducted by Almeida and Amorim [36] showed that the methanolic extract of the root of* C. infestus* Pax & K. Hoffm presented relevant antioxidant activity and phenolic compounds in the species, suggesting that* C. quercifolius*, due to phenols in its composition, showed antioxidant activity. However, Conforti et al. [37] have demonstrated that there is still controversy between the correlation of antioxidant activity and the content of phenolic compounds of plant extracts.

Studies have shown that plants used in traditional medicine have the effect of prevention and protection under conditions of oxidative stress [38–40]. The improvement of the conditions of oxidative stress by plants has been associated with phenolic compounds such as flavonoids and other polyphenols. These bioactive molecules exhibit a variety of biological effects, such as antithrombotic, anti-inflammatory, and anticancer effects, as a result of their antioxidant properties [41].

Table 2 shows the results of antimicrobial activity of methanolic extracts of* C. quercifolius* against Gram-positive and Gram-negative bacteria and fungi. By agar diffusion method, the LME and the RBME showed similar activity by inhibiting the growth of* Enterococcus faecium*,* Enterococcus faecalis*,* Staphylococcus epidermidis*, and* Pseudomonas aeruginosa*, while the RME inhibited only the strain of* Staphylococcus epidermidis*. There was no inhibitory response from the extracts tested against* Escherichia coli* and* Klebsiella pneumoniae* strains, thus demonstrating that the methanolic extracts of faveleira showed better inhibition against strains of Gram-positive bacteria. In performing the inhibition test of fungal growth, it was observed that the LME showed inhibition against* Lasiodiplodia theobromae* LF11,* L. theobromae* LF124, and* Colletotrichum gloeosporioides* LF50, whereas RME and RBME showed inhibition only against* Colletotrichum gloeosporioides* LF50.

Antimicrobial assays against bacterial strains and fungi tested, using commercial drugs (Chloramphenicol, Streptomycin, and Thiabendazole) as a positive control, showed no growth of microorganisms.

The MIC was studied for bacterial strains that were sensitive to the methanolic extracts of faveleira in the agar diffusion method (Table 2). For Holetz et al. [42], plant extracts have a good antimicrobial activity when they feature a MIC less than 100 *μ*g·mL^−1^, moderate activity with a MIC of 100 to 500 *μ*g·mL^−1^, and weak activity with a MIC of 500 to 1000 *μ*g·mL^−1^ and are inactive when the MIC exceeds 1000 *μ*g·mL^−1^. According to the results, LME, RME, and RBME from* C. quercifolius* presented a good antibacterial activity.

Peixoto Sobrinho et al. [26] evaluated the antibacterial activity of the methanolic extract from the bark and leaf of* C. quercifolius*. In the agar diffusion method, the extracts showed activity against strains of* Staphylococcus* and* E. faecalis* and showed no activity against* P. aeruginosa*,* K. pneumoniae*, and* E. coli*. In determining the MIC, the leaf extract was inactive against the* Staphylococcus* strains, while the bark extract was active with a MIC of 250 to 500 *μ*g·mL^−1^. In the present study, the methanolic extracts of the leaf, root, and root bark of* C. quercifolius* were active against* Staphylococcus* and* E. faecalis strains*, where the root bark extract presented the lowest value of the MIC (15.62 *μ*g·mL^−1^), being the most active for* Staphylococcus*. Almeida [43] performed an antimicrobial assay with strains of bacteria and fungi using hydroalcoholic extract of the leaves of* C. quercifolius*, obtaining MIC and MFC values that exceeded 2000 *μ*g·mL^−1^.

Ahmad and Beg [44] believe that the antimicrobial effect of vegetal extracts is largely due to the presence of flavonoids in their composition, thus corroborating the results found for the* C. quercifolius *species.

In the evaluation of fungicidal activity of the extracts of faveleira, the RME and RBME only inhibited the growth of* Colletotrichum gloeosporioides*, of seven fungi tested, while the LME extract exhibited good activity against* Lasiodiplodia theobromae* LF11,* L. theobromae* LF124, and* Colletotrichum gloeosporioides* (Table 2).

In a study conducted with the oil from the seeds and extract from the leaves of the faveleira, Ribeiro [45] found reduced mycelial growth and germination of conidia of* F. oxysporum* f. sp.* tracheiphilum*. This is a different outcome from the findings of this study, for none of the methanolic extracts of the faveleira exhibited growth inhibition of fungi of the same family.

The bioautography method employing Ellman reagent allows for the identification of compounds capable of inhibiting acetylcholinesterase (AChE) in extracts, which enables a fractionation targeted to the isolation of those compounds. It is important to note that this method only assists in optimizing the selection and does not eliminate, under any circumstance, the* in vivo* tests [24].

**Figure 1:**
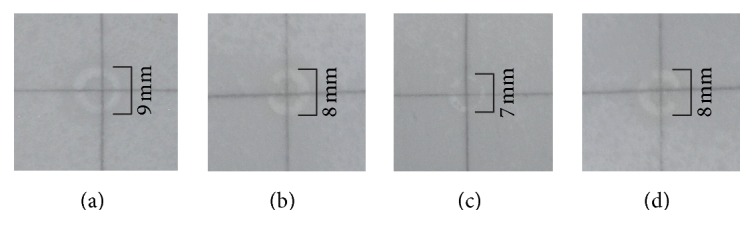
Halos of inhibition of acetylcholinesterase enzyme by the methanolic extracts of* C. quercifolius*. (a) Eserine; (b) LME (leaves); (c) RME (root); (d) RBME (root bark).

As shown in Figure 1, the faveleira extracts showed halo formation in the inhibition test of AChE, with measurements of 8 mm for LME, 7 mm for RME, and 8 mm for the RBME, while the standard featured a halo of 9 mm.

**Figure 2:**
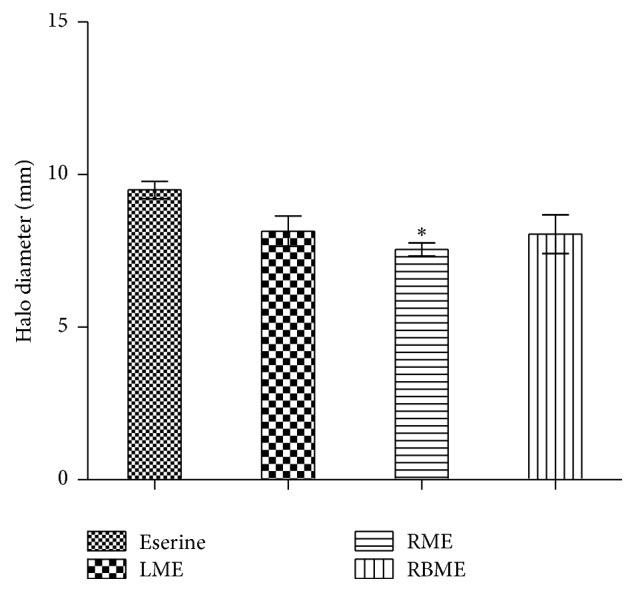
Result of inhibition of the acetylcholinesterase enzyme from methanolic extracts of* C. quercifolius*: LME (leaves); RME (root); RBME (root bark). To analyze the significance of the differences between the samples the authors used Variance Analysis (ANOVA) followed by the Newman-Keuls comparison test, *p* < 0.05 being considered significant. *∗* indicates a significant difference.

All the methanolic extracts of the faveleira presented anticholinesterase activity. There was no statistically significant difference between the LME and RBME with the reference drug (Eserine), which represents a new source of bioactive compounds with potential as acetylcholinesterase inhibitor (Figure 2).

Orhan et al. [46], Adsersen et al. [47], Ingkaninan et al. [48], and Mukherjee et al. [49] describe the ability of several plants to inhibit the enzyme AChE, demonstrating a great therapeutic potential in the treatment of Alzheimer's disease and other neurodegenerative maladies. The identification of these plants, through phytochemical and ethnopharmacological studies, provides the possibility of discovery and development of new drugs that could be relevant for the treatment of degenerative diseases of the nervous system, through the effect of AChE inhibition [49, 50].

One of the first AChE inhibitors used was physostigmine, an alkaloid isolated from a plant of the Leguminosae family, although this alkaloid is no longer used clinically, due to its short half-life. Through the analysis of their chemical structure new inhibitors, such as rivastigmine, were developed and approved as a therapeutic possibility of the treatment of patients with Alzheimer's disease [49]. Despite the negative presence of alkaloids in the faveleira extracts tested, the plant presented satisfactory activity of enzyme inhibition, when compared with the reference substance, raising the possibility that new untested substances may act on the inhibition of AChE.

The use of less complex animal organisms, as is the case of* Artemia salina*, can be done to evaluate the toxicity of bioactive compounds, besides being a simple, fast, and low-cost test. Several studies have shown the use of* Artemia salina* to evaluate the toxicity of extracts, of isolated and purified substances of medicinal plants [31, 51–54], thus decreasing the use of animals for experimentation.

In the evaluation of toxicity of methanolic extracts of* C. quercifolius* against nauplii of* Artemia* sp. (Table 3), the results revealed that RME was the one showing the highest toxicity LC_50_ = 84.76 *μ*g·mL^−1^, followed by RBME with LC_50_ = 341.45 *μ*g·mL^−1^ and LME with LC_50_ = 1079.78 *μ*g·mL^−1^. Souza [55] describes that the absence of toxicity can be an advantage for the possibility of using isolated substances in developing herbal medicines for use in humans.

Meyer et al. [25] make a correlation of toxicity against* A. salina* with possible antitumor activity, as well as antifungal, virucidal, antimicrobial, trypanosomicide, and parasiticide activity. McLaughlin et al. [56] make a comparison of the toxicity found on this microcrustacean with a good cytotoxic activity against human tumors.

In a test of toxicity against* Artemia salina*, conducted by Fabri et al. [57], with a plant of the same family as the faveleira, the methanolic extract of the leaves was used and produced LC_50_ significantly toxic of 15.6 *μ*g·mL^−1^. For those authors, the toxicity of a substance is weak when the LC_50_ is above 250 *μ*g·mL^−1^ so, from the results of the present study we can assume that the LME and the RBME have a low toxicity, whereas the RME showed a significant toxicity.

## 4. Conclusions

The present study, of crude methanolic extracts of* Cnidoscolus quercifolius* Pohl, has shown promising results in all tests performed. Standing out among these auspicious outcomes are the antioxidant effects, the bactericidal and fungicidal potential, and the inhibitory effect against the acetylcholinesterase enzyme in two extracts tested, which showed no significant difference when compared with the reference drug. Thus, we can infer that further testing will be needed, through fractionation of extracts and evaluation of their toxicity, in order to isolate and characterize which bioactive compounds are responsible for the findings, and establish the safe and effective dosage and, additionally, to verify the possibility of its use in the prevention and cure of diseases, contributing to improve the health of the population through increased access to herbal remedies and medicinal plants and their rational use, based on the sustainable development and management of biodiversity in the state of Ceará, considering the inherent social, ethical, economic, and ecological aspects.

## Figures and Tables

**Table 1:** Preliminary phytochemical analysis of *C. quercifolius* Pohl methanolic extracts.

Class of metabolites	LME	RME	RBME
Phenols and tannins	+	+	+
Anthocyanins and anthocyanidins	−	−	−
Flavones, flavonols, xanthones, flavanones	+	+	+
Chalcones and aurones	−	−	−
Leucoanthocyanidins	−	−	−
Catechins (catechin tannins)	−	−	−
Steroids and triterpenoids	−	−	−
Saponins	−	−	−

+: presence; −: absence; LME: leaf methanolic extract; RME: root methanolic extract; RBME: root bark methanolic extract.

**Table 2:** Antimicrobial activity of the methanolic extracts of *C. quercifolius *Pohl against 13 microorganisms.

Microorganisms	LME			RME			RBME		
	Disk diffusion	MIC (*µ*g·mL^−1^)	MBC (or MFC) (*µ*g·mL^−1^)	Disk diffusion	MIC (*µ*g·mL^−1^)	MBC (or MFC) (*µ*g·mL^−1^)	Disk diffusion	MIC (*µ*g·mL^−1^)	MBC (or MFC) (*µ*g·mL^−1^)
*Enterococcus faecium* ATCC 51559	*+*	62,5	62,5	−	−	−	+	31,25	31,25
*Pseudomonas aeruginosa* ATCC 10145	*+*	7,81	7,81	−	−	−	+	31,25	31,25
*Enterococcus faecalis* ATCC 19433	*+*	62,5	62,5	−	−	−	+	62,5	62,5
*Escherichia coli *ATCC 11775	*−*	−	−	−	−	−	−	−	−
*Staphylococcus epidermidis* ATCC 12228	*+*	31,25	31,25	**+**	7,81	7,81	**+**	15,62	15,62
*Klebsiella pneumoniae *ATCC 13883	*−*	−	−	−	−	−	−	−	−

*Fusarium* sp. LF48	ND	−	−	ND	−	−	ND	−	−
*F. solani* LF04	ND	−	−	ND	−	−	ND	−	−
*F. solani* LF104	ND	−	−	ND	−	−	ND	−	−
*Lasiodiplodia theobromae* LF11	ND	31,25	31,25	ND	−	−	ND	−	−
*L. theobromae* LF124	ND	31,25	31,25	ND	−	−	ND	−	−
*L. theobromae* LF126	ND	−	−	ND	−	−	ND	−	−
*Colletotrichum gloeosporioides* LF50	ND	62,5	62,5	ND	15,62	15,62	ND	62,5	62,5

ATCC: American Type Culture Collection; LF: Laboratory of Phytopathology; MIC: Minimum Inhibitory Concentration; MBC: Minimum Bactericidal Concentration; MFC: Minimum Fungicidal Concentration; +: inhibition; −: no inhibition; ND: not determined.

**Table 3:** *Brine shrimp* lethality test of the methanolic extracts from *C. quercifolius*.

Samples	LC_50_ (*μ*g·mL^−1^)	Confidence intervals	
		Lower	Upper
LME	1079,78	709,85	1642,48
RME	84,76	47,7	150,61
RBME	341,45	293,62	397,08

LC_50_: lethal concentration that kills 50% of larvae; LME (leaves); C: RME (root); D: RBME (root bark).
